# Application of NMRK immobilized enzymes in the synthesis of NMN

**DOI:** 10.1371/journal.pone.0335646

**Published:** 2026-02-19

**Authors:** Shan Yang, Ting Xiang, Jinchun Qian, Wei Sun, Wenjun Liu, Nengwen Yi, Zheng-Hong Qin

**Affiliations:** 1 Medical Department, Suzhou Gao Bo Vocational College, Suzhou, China; 2 Department of Pharmaceutical Management, Suzhou Gao Bo Vocational College, Suzhou, China; 3 Institute of Health Science and Technology, Suzhou Gao Bo Vocational College, Suzhou, China; 4 Suzhou Ren Ben Pharmaceutical Co., Ltd., Suzhou, China; Texas A&M University, UNITED STATES OF AMERICA

## Abstract

The purpose of the study is to provide a method for mass production of nicotinamide mononucleotide (NMN) using an immobilized nicotinamide riboside kinase (NMRK). We synthesized a gene sequence comprising the gene encoding the enzyme. At the 3’ end of this NMRK-encoding gene, an optimized cellulose-binding domain (CBD) sequence was linked via a linker-encoding gene segment.Using the pET-32a plasmid as the vector and Escherichia coli BL21(DE3) as the host cell, the recombinant E. coli strain BL21/pET-32a-nmrk-1 was constructed and cultivated. This recombinant strain BL21/pET-32a-nmrk-1 secreted a significantly higher yield of the NMRK enzyme protein (32 mg/mL), and the secreted recombinant NMRK enzyme exhibited superior activity (80 U/g). Activated microcrystalline cellulose was mixed with the crude enzyme solution containing the recombinant NMRK enzyme. Following processing, the immobilized NMRK enzyme was obtained. This immobilized enzyme catalyzed the synthesis of NMN from 0.07 mol/L nicotinamide riboside (NR), achieving a conversion rate of 90% and a purity of 33%. The recombinant NMRK enzyme retained good activity after immobilization onto the carrier, enabling its reuse for more than 10 cycles. The initial activity of the immobilized NMRK enzyme was 540 U/g. After 11 cycles of use, the activity was 390 U/g, representing only a 27.8% decline in activity. This approach enhances the utilization efficiency of the NMRK enzyme while simultaneously reducing the production cost of NMN.

## Introduction

Nicotinamide Adenine Dinucleotide (NAD+), also known as coenzyme I, is ubiquitously distributed in all human cells. It participates in thousands of biocatalytic reactions and is an essential coenzyme for life [1]. NMN is a naturally occurring bioactive nucleotide. As a precursor to NAD + , it is extensively involved in numerous biochemical reactions in the human body and is closely linked to immunity, metabolism, and other vital functions [2,3].

Research indicates that with increasing age, levels of both NMN and NAD+ in the human body exhibit a declining trend, while the NAD+ metabolite nicotinamide (NAM) shows an increasing trend [4]. This decline in NAD + is recognized as a primary factor contributing to the onset of diseases and disabilities such as hearing and vision loss, cognitive and motor dysfunction, immune deficiency, and disorders stemming from dysregulated immune and inflammatory responses (e.g., arthritis, metabolic disorders, and cardiovascular diseases) [5–9]. Studies have shown that supplementing mice with NAD+ precursors to elevate NAD+ levels in their tissues can alleviate metabolic syndrome, enhance cardiovascular function, improve neurodegeneration, and increase muscle contractile strength [10]. Further research demonstrates that NMN can be directly absorbed by intestinal cells, whereas NAD + , being a large molecule, cannot [11–13]. Consequently, it is currently believed that supplementing with NMN is crucial for increasing NAD+ levels in humans, thereby delaying, ameliorating, or preventing diseases and disabilities associated with declining NAD + , and ultimately maintaining human health [14]. Studies indicate that long-term NMN supplementation in aged mice enhances physical activity, increases energy expenditure, and reduces weight gain [15]. In recent years, the demand for NMN in the nutraceutical market has also increased annually [16].

Currently, NMN is primarily synthesized via chemical methods or enzymatic methods. Chemical synthesis typically uses nicotinamide as a starting material and involves multiple reaction steps. This approach suffers from several drawbacks: a long reaction pathway, harsh reaction conditions, poor selectivity, tendency for byproduct formation, low product purity, low yield, the requirement for expensive reagents, and high costs. Furthermore, it necessitates the use of large volumes of organic solvents, leading to environmental pollution [17]. Therefore, this route is unsuitable for large-scale industrial production. The enzymatic method utilizes crude enzyme solutions obtained from the fermentation of genetically engineered bacteria secreting the NMRK enzyme to catalyze the synthesis of NMN from NR [18]. Compared to the chemical method, the enzymatic approach offers advantages such as a shorter reaction pathway, milder reaction conditions, and being more environmentally friendly and safe [19]. However, NMRK enzyme activity is susceptible to inhibition by NR concentration. Typically, the crude enzyme solution can only catalyze NR once before needing replacement, resulting in substantial raw NMRK consumption. Moreover, it can only be used to catalyze low concentrations of NR, severely limiting NMN yield and production efficiency [20]. Consequently, this process route is also unsuitable for large-scale industrial production. There is an urgent need to enhance the yield and production efficiency of NMN synthesis via the enzymatic method to achieve large-scale industrial production of NMN.

In this study, we synthesized a gene comprising a gene encoding NMRK enzyme, and the 3’ end of the gene encoding NMRK enzyme is connected with an optimized CBD sequence by the gene encoding linker.The synthesized gene and the pET-32a plasmid were digested using the restriction endonucleases Bacillus amyloliquefaciens H(BamHI) and Xanthomonas holcicola (XhoI), followed by ligation to generate the recombinant plasmid. This ligation product was then transformed into E.coli BL21(DE3). After cultivation, the recombinant strain BL21/pET-32a-nmrk-1 was obtained. This strain demonstrated enhanced secretion of the NMRK enzyme with superior protein yield and enzymatic activity.The activated microcrystalline cellulose was mixed with the crude enzyme solution containing recombinant NMRK enzyme, and immobilized at pH 5 ~ 9, temperature 4 ~ 10°C, and rotation speed 50 ~ 200 r/min for 2 ~ 6 h. NMN was synthesized by using immobilized NMRK enzyme to catalyze NR at pH 4 ~ 6, temperature 25 ~ 40°C, and rotation speed of 150 ~ 250 r/min for 2 ~ 4 h. The improvement of the binding stability between NMRK enzyme and immobilized vector can effectively prevent NMRK enzyme from falling off the immobilized carrier during use, and realize the repeated use of NMRK enzyme. The method of preparing immobilized NMRK enzyme and synthesizing NMN provided in this study improve the efficiency of enzyme use, simplify the purification process of products, improve the purity of products, and reduce the production cost of NMN, which is of great significance for the production of NMN.

## Materials and methods

### Plasmid construction

E.coli BL21(DE3) is used as the host cell. Carrier PET-32a is used for protein expression. This study provides a study gene consisting of a gene encoding NMRK enzyme, a gene encoding a linker, and an optimized CBD sequence. The CBD sequence is linked at the 3’ end of the gene encoding the NMRK enzyme by a gene encoding a linker. synthesize 1 gene (Bam HI. + research gene + Xho I.); Synthesis of 2 genes (Bam HI. + gene encoding NMRK enzyme + Xho I.). All genes are provided by Shandong Lankang Pharmaceutical Co., Ltd.,. The synthetic 1 gene of E.coli BL21(DE3) was constructed on PET-32a, and the plasmid PET-32a-nmrk-1 was obtained. The synthetic 2 genes of E.coli BL21(DE3) were constructed on PET-32a to obtain the plasmid PET-32a-nmrk-2.

### Plasmid construction

The existing pET32a-CBD vector was linearized by double digestion using the restriction enzymes HindIII and XhoI. The linearized pET32a-CBD vector was then assembled with the Linker fragment and the NMRK2 fragment via homologous recombination, successfully constructing the pET32a-CBD-NMRK2 recombinant vector. During the process, a 1% agarose gel was prepared. Samples of the original pET32a-CBD vector, the double-digested pET32a vector product, the annealed Linker fragment, and the PCR-amplified NMRK2 fragment were loaded into the gel wells. Electrophoresis was performed at 110V for 30 minutes. Following electrophoresis, the results were visualized using a gel imaging system, and the target DNA bands were excised for recovery. DNA fragment extraction was performed strictly according to the instructions of the Axygen DNA Gel Extraction Kit. Finally, the purified NMRK2 target fragment and Linker fragment were ligated with the linearized pET32a-CBD vector. The pET-32a-nmrk-2 plasmid was constructed using an identical strategy and procedure. The specific primer sequences used are detailed in Table 1.

**Table 1:** Primers used in this study.

Name	Primer sequence (5′–3′)
Linker-F	TGGGGTAAAGAACCGAAGCTTGGTGGCGGTGGCTCTGGCGGTGGTGGGAGTGGAGGTGGGGGATCACTGGAAGTTCTGTTCCAGGGGCCC
Linker-R	GGGCCCCTGGAACAGAACTTCCAGTGATCCCCCACCTCCACTCCCACCACCGCCAGAGCCACCGCCACCAAGCTTCGGTTCTTTACCCCA
NMRK2-F	AAGTTCTGTTCCAGGGGCCCGAGCTCATGAAGCTGATTGTTGGTATT
NMRK2-R	CAGTGGTGGTGGTGGTGGTGCTCGAGTTACATACTATCTTGCTGGC

### Construction of recombinant E. coli BL21(DE3)

The constructed plasmids pET-32a-nmrk-1 and pET-32a-nmrk-2 were co-transformed into the expression host E.coli BL21(DE3), generating recombinant strains designated BL21/pET-32a-nmrk-1 and BL21/pET-32a-nmrk-2. Transformed cells were plated on LB agar medium containing 15 mg/L ampicillin and incubated upside-down at 37°C for 12 h. Single transformant colonies were then inoculated into 5 mL LB liquid medium supplemented with 15 mg/L ampicillin and cultured in shake flasks at 37°C with 180 rpm agitation for 12 h. Plasmids were extracted from these cultures and validated through both restriction enzyme digestion and DNA sequencing. Confirmed correct constructs yielded the final recombinant E. coli strains BL21/pET-32a-nmrk-1 and BL21/pET-32a-nmrk-2, which were preserved at −80°C in 25% glycerol.

### Preparation of recombinant NMRK enzyme

A single colony of recombinant E. coli BL21/pET-32a-nmrk-1 was inoculated into 5 mL of seed medium (containing 15 mg/L ampicillin, 12 g/L yeast extract, 24 g/L peptone, 16.43 g/L K₂HPO₄, 2.31 g/L KH₂PO₄, and 4 mL/L glycerol) and incubated at 37°C with 180 rpm agitation for 12 h to obtain the primary seed culture. This culture was transferred at 2% (v/v) inoculum into 500 mL of identical ampicillin-supplemented seed medium and cultivated at 37°C, 220 rpm for 8 h to prepare the secondary seed culture. The entire secondary culture was then transferred to a 5-L fermenter containing 3 L of fermentation medium (8 g/L yeast extract, 12 g/L peptone, 10 g/L K₂HPO₄·3H₂O, 5 g/L NaCl, 2.5 g/L (NH₄)₂SO₄, 2.1 g/L citric acid monohydrate, 0.3 g/L ferric ammonium citrate, 5 g/L glycerol, 0.05% w/w polyether GRE antifoam, pH adjusted to 7.0 with 20% w/w ammonia), supplemented with 100 mL of 20 g/L anhydrous MgSO₄ solution. Fermentation proceeded at 37°C, 500 rpm, and 1 vvm aeration for 12 h until OD₆₀₀ reached 25 (with pH maintained at 6.9 via fed-batch fermentation medium). After adding IPTG to 1 mmol/L final concentration, fermentation continued at 25°C, 500 rpm, 1 vvm for 10 h until OD₆₀₀ = 35. The broth was centrifuged at 10,000 rpm for 10 min, and the cell pellet (100 g wet weight) was resuspended in 500 mL distilled water. The suspension was disrupted twice using a high-pressure homogenizer at 900 MPa, followed by centrifugation (10,000 rpm, 10 min). The supernatant was adjusted to pH 7.0 with 1 mol/L KOH, treated with 1% w/w chitosan solution containing 5% w/w anhydrous CaCl₂ until pH 5.0, then readjusted to pH 7.5 with 1 mol/L KOH to induce flocculation. After final centrifugation (10,000 rpm, 10 min), the supernatant was collected as the crude recombinant NMRK enzyme solution.Protein concentration of the crude enzyme solution was determined using UV spectrophotometry and the Coomassie Brilliant Blue method. NMRK enzyme activity was assayed according to Chinese industry standard SB/T 10317−1999 as follows: The 100-mL reaction system contained 0.12 mol/L NR, 15 mmol/L MgSO₄·7H₂O, 0.13 mol/L Adenosine Triphosphate (ATP), 0.2 mol/L PBS buffer (pH 8.0), and 10 g immobilized NMRK enzyme. After homogenization, the reaction proceeded at 30°C with 200 rpm agitation. Samples were withdrawn hourly for 10 h, and NMN production was quantified via HPLC at 260 nm. Enzyme activity (U) was calculated from the slope (ΔS) of the initial linear phase of the peak area-time plot, where one unit is defined as the amount of enzyme producing 1 μmol NMN per hour at 30°C. Activity was calculated using the formula:


$$\text{U}=\frac{△\text{S}\times \text{V}}{\text{S}\times \text{334}\times \text{m}}\times {\text{10}}^{\text{6}}$$

△S represents the change in NMN peak area at 280 nm, S is the peak area corresponding to a 1 g/mL NMN standard,V is the enzymatic reaction volume in mL. 334 g/mol is the molecular weight of NMN. m denotes the mass (g) of NMRK added to the system.

**Fig 1:**
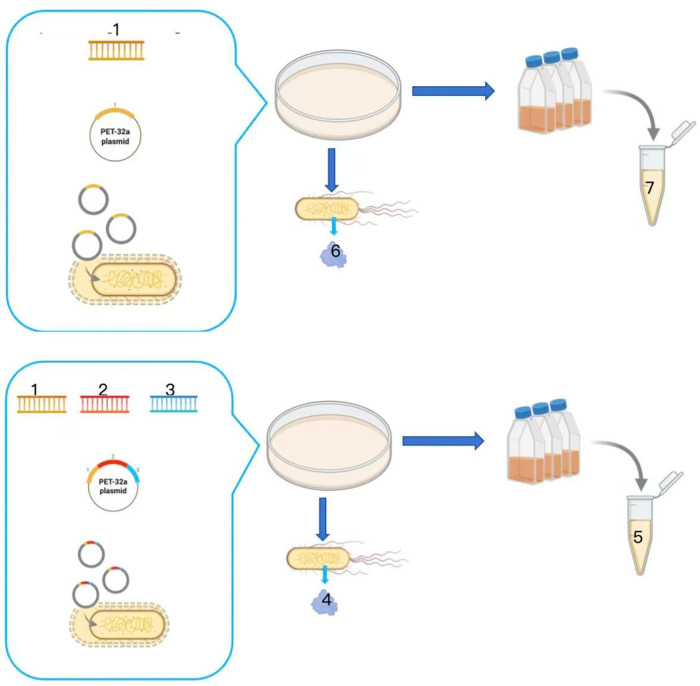
Schematic of NMRK fusion construct and enzyme preparation. 1: Gene encoding NMRK enzyme; 2: Gene encoding linker; 3: Optimized CBD sequence; 4: Recombinant NMRK enzyme; 5: Crude enzyme solution of recombinant NMRK; 6: Wild-type NMRK enzyme; 7: Crude enzyme solution of wild-type NMRK.

The same procedure was applied to process a single colony of recombinant E. coli BL21/pET-32a-nmrk-2, yielding a crude enzyme solution containing the wild-type NMRK enzyme. Both crude enzyme solutions (from BL21/pET-32a-nmrk-1 and BL21/pET-32a-nmrk-2) were then subjected to measurements of protein concentration and NMRK enzyme activity.Schematic of NMRK fusion construct and enzyme preparation is shown in Fig 1.

### Preparation of immobilized NMRK enzyme

The immobilized NMRK enzyme was prepared through the following steps: 1.Microcrystalline cellulose (Sinopharm Group) was activated by immersion in 2% (w/w) glutaraldehyde solution for 1 h at room temperature, followed by filtration through a 5 µm membrane. 2.The activated cellulose was washed with 500 mL of 0.2 mol/L Na₂HPO₄ solution, then mixed with crude recombinant NMRK enzyme solution at a 5:1 mass ratio (cellulose:enzyme). This mixture was combined with 0.2 mol/LNa₂HPO₄ solution at 1:1 volume ratio, adjusted to pH 8.0 with 1 mol/L KOH, and reacted at 10°C with 100 rpm agitation for 4 h. The crude immobilized enzyme was collected by 5 μm filtration. 3.Purification involved sequential washing with 500 mL 0.2 mol/L Na₂HPO₄, treatment with 500 mL 0.2 mol/L Na₂HPO₄ containing 0.5 mol/L NaCl (150 rpm, 50 min), final filtration, additional Na₂HPO₄ washing, and vacuum drying to yield Immobilized NMRK Enzyme A.

Using the identical procedure, immobilized NMRK enzymes B, C, D, and E were prepared by modifying reaction conditions. Comparative synthesis conditions for each immobilized NMRK enzyme group are presented in Table 2.

**Table 2:** Comparison of synthesis reaction conditions for immobilized NMRK enzymes.

Group	Immobilized NMRK Enzyme A	Immobilized NMRK Enzyme B	Immobilized NMRK Enzyme C	Immobilized NMRK Enzyme D	Immobilized NMRK Enzyme E
Enzyme Source	Crude enzyme solution of immobilized NMRK	Crude enzyme solution of immobilized NMRK	Crude enzyme solution of immobilized NMRK	Crude enzyme solution of immobilized NMRK	Crude enzyme solution of wild-type NMRK
Carrier Material	Microcrystalline cellulose	Microcrystalline cellulose	LX-1000NH	LX-1000HFA	Microcrystalline cellulose
PH	8.0	5.0	8.0	8.0	5.0
Temperat-ure (°C)	10	5	10	10	5
Glutarald-ehyde Solution (w/w)	2%	1%	2%	2%	1%

Table notes the different reaction conditions for the preparation of immobilized NMRK enzymes B, C, D, and E.

### Impact of enzyme and immobilization carrier selection on immobilized enzyme performance

To evaluate the impact of enzyme and carrier selection on immobilized enzyme performance, the following experimental protocol was implemented: A 0.05 mol/L NR solution was prepared, supplemented with ATP to a final concentration of 0.06 mol/L to form the substrate solution. Immobilized NMRK enzymes A through E were separately introduced into this substrate solution at 20 g/L final concentration. After pH adjustment to 5.5 using 6 mol/L NaOH, the reaction systems were incubated at 30°C with 180 rpm agitation for 2 hours, yielding NMN-containing reaction mixtures A-E. Subsequent HPLC analysis followed Chinese Pharmacopoeia (2010 Ed.) Part II Appendix VD specifications, employing a ReproSil-Pur 120 ODS-3 column (5 µm, 4.6 × 250 mm) with methanol (mobile phase A) and 100 mmol/L NaH₂PO₄ aqueous solution (mobile phase B). Separation was achieved through the Table 3 defined gradient program under standardized conditions (20 µL injection volume, 25°C column temperature, 1.0 mL/min flow rate, 260 nm detection wavelength). NMN purity and conversion rates in reaction mixtures A-E were calculated via the area normalization method using HPLC system software.

**Table 3:** Gradient elution program.

Elution time/min	A (%)	B (%)
**0**	5	95
**10**	8	92
**15**	15	85
**21**	15	85
**21.10**	5	95

The table shows the gradient elution of NMN-containing reaction solution A ~ E.

### Synthetic NMN

NMN synthesis was performed across four NR concentrations (0.05,0.07,0.09,0.12) mol/L with stoichiometrically matched ATP supplementation (0.06,0.08,0.10,0.13) mol/L. Each concentration set reacted separately with either immobilized NMRK Enzyme A or B (20 g/L), while wild-type NMRK crude enzyme served as a non-immobilized control exclusively at 0.05 mol/L NR. All reactions proceeded at 30°C with 180 rpm agitation for 2 h following pH adjustment to 5.5. NMN purity and conversion rates were determined for the resultant nine reaction systems.

### Impact of NR catalysis on activity retention in immobilized NMRK enzymes

To investigate the impact of NR catalysis on activity retention in immobilized NMRK enzymes, the following experimental procedure was implemented: Activity assay of immobilized NMRK enzyme: A 100-mL reaction system containing 0.12 mol/L NR, 15 mmol/L MgSO₄·7H₂O, 0.13 mol/L ATP, 0.2 mol/L PBS buffer (pH 8.0), and 10 g immobilized NMRK enzyme was homogenized and incubated at 30°C with 200 rpm agitation. Samples were withdrawn hourly over 10 h, and NMN production was quantified via HPLC at 260 nm. The assay solution prepared according to the above system was homogenized by agitation, then reacted at 30°C and 200 rpm. Samples were taken every 1 h, and the NMN peak area was measured via HPLC at 260 nm for 10 consecutive hours. Peak area was plotted against time, the ΔS value was calculated from the initial linear phase of the reaction, and enzyme activity was computed according to the formula provided earlier.

Post-catalysis activity assessment: The immobilized enzyme was reused for 11 consecutive NR-to-NMN conversion cycles. After each cycle, the enzyme was recovered by 5 µm membrane filtration and subjected to identical activity measurement before subsequent use.

## Results

### Comparative analysis of protein concentration and NMRK activity in crude enzyme solutions from immobilized and wild-type enzymes

**Fig 2:**
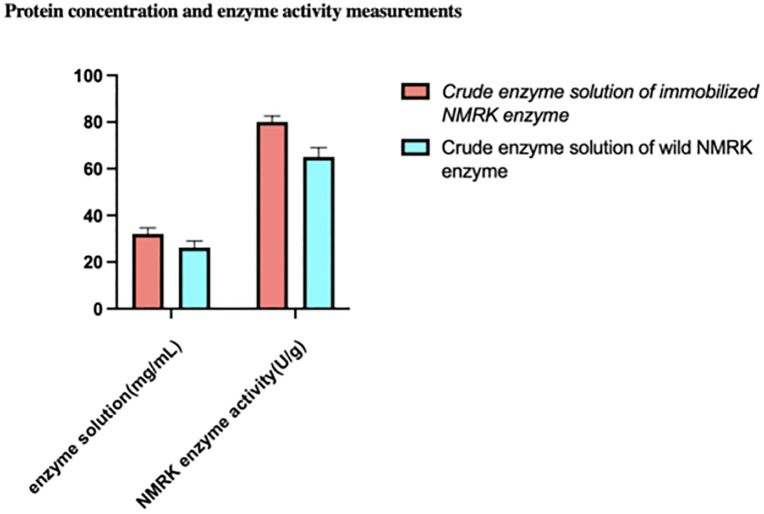
Results of protein concentration and NMRK enzyme activity in two crude Enzyme extracts.

Crude enzyme solutions derived from immobilized NMRK enzyme and wild-type NMRK enzyme were subjected to protein concentration and enzymatic activity assays. A single colony of recombinant E. coli BL21/pET-32a-nmrk-1 was processed to yield crude enzyme solution containing the soluble NMRK fusion enzyme (pre-immobilization). Similarly, a single colony of recombinant E. coli BL21/pET-32a-nmrk-2 was processed to generate crude enzyme solution containing wild-type NMRK enzyme. Protein concentrations and NMRK activities of both crude enzyme solutions were measured, with results presented in Fig 2.

**Fig 3:**
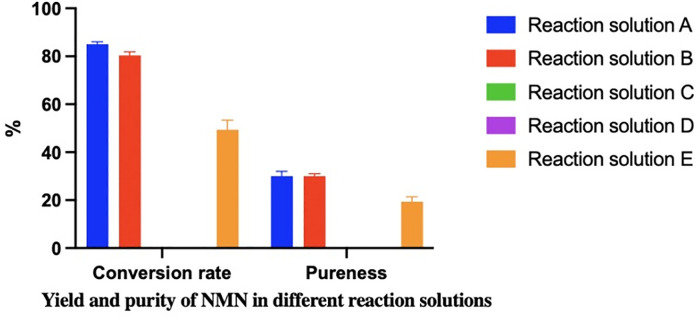
NMN yield and purity from biocatalysis using immobilized NMRK enzymes A-E.

Fig 3 presents the yield and purity measurements of NMN in different reaction mixtures. Both yield and purity were 0% in reaction mixtures using Immobilized NMRK Enzymes C and D, indicating these variants failed to catalyze NR-to-NMN conversion. This demonstrates that immobilizing recombinant NMRK enzyme onto LX-1000NH and LX-1000HFA carriers resulted in complete enzyme inactivation. In contrast, Immobilized NMRK Enzyme E exhibited catalytic capability for NMN synthesis, while Immobilized NMRK enzymes A and B demonstrated superior catalytic efficiency to enzyme E. These results confirm that the enzyme selection (recombinant NMRK) and carrier material (microcrystalline cellulose) used for Enzymes A-B effectively preserved NMRK activity. Comparative analysis establishes that combining recombinant NMRK with microcrystalline cellulose delivers enhanced efficacy over wild-type NMRK immobilized on LX-1000NH or LX-1000HFA.

### Measurement of NMN yield and purity synthesized at different NR concentrations

**Fig 4:**
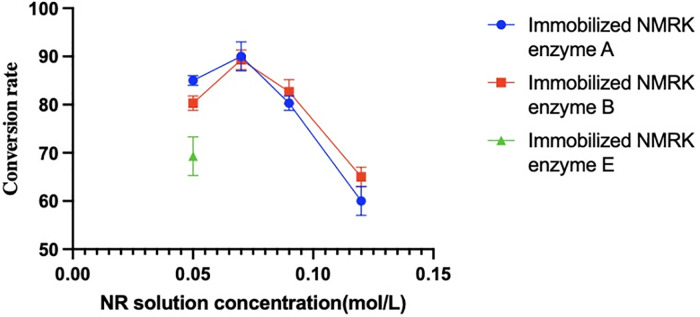
NMN yield at different NR concentrations.

**Fig 5:**
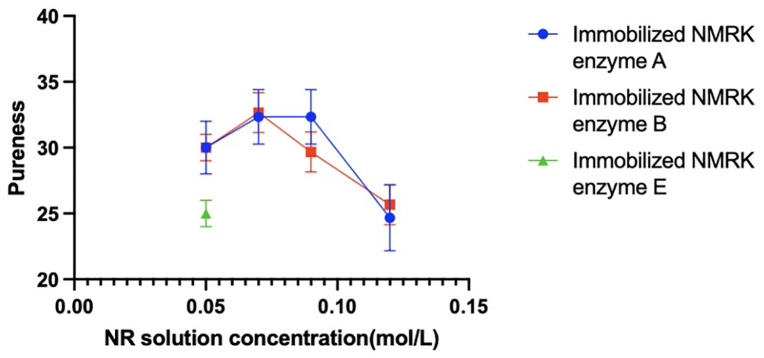
NMN purity at different NR concentrations.

Fig 4 shows the NMN yields synthesized using immobilized NMRK enzymes A-B and wild-type NMRK crude enzyme extracts at different NR concentrations, while Fig 5 presents corresponding purity measurements. The crude enzyme extract containing wild-type NMRK catalyzed NR-to-NMN conversion. Immobilized NMRK enzymes A-B also effectively catalyzed this reaction, demonstrating superior catalytic performance compared to the wild-type crude enzyme extract. This indicates that both enzyme selection and immobilization carrier choice effectively preserve NMRK activity. Furthermore, immobilized enzymes A and B successfully catalyzed NMN synthesis across all tested NR concentrations, demonstrating that this enzyme-immobilization carrier combination enhances NMRK stability. Consequently, the catalytic efficiency of immobilized enzymes A-B exhibits significantly greater resistance to inhibition by increasing NR concentrations.

**Fig 6:**
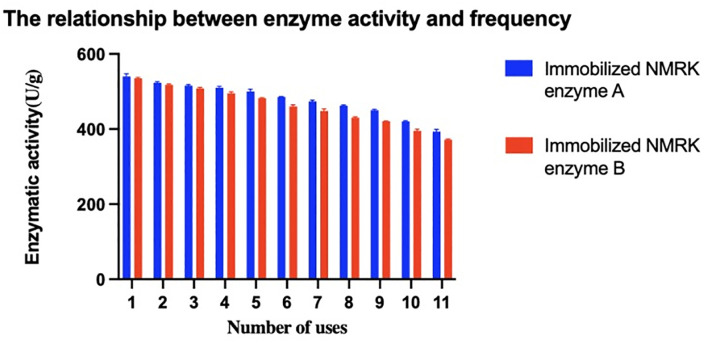
Activity of immobilized NMRK enzymes (U/g).

**Fig 7:**
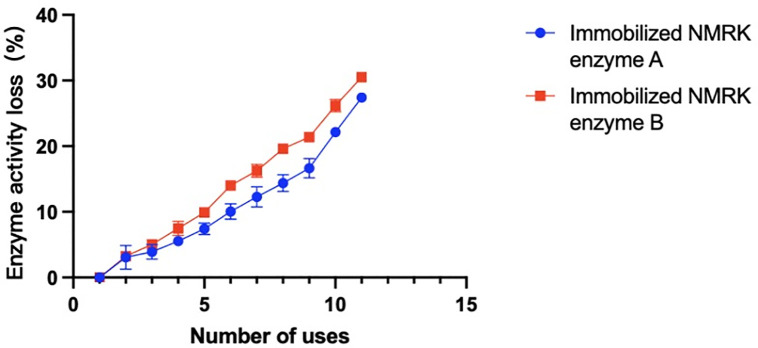
Activity reduction percentage of reused immobilized NMRK enzymes.

Fig 6 shows the impact of NR catalysis on immobilized enzyme activity, with post-reaction activity reduction rates presented in Fig 7. After 11 reuse cycles for NMN synthesis, immobilized NMRK enzymes A and B maintained high activity levels with only 27.7% and 30.5% activity reduction respectively. This further demonstrates that combining recombinant NMRK with microcrystalline cellulose carriers effectively preserves enzymatic activity, enhances enzyme stability, and improves binding stability between the enzyme and carrier. Crucially, enzyme reuse significantly reduces production costs while ensuring efficient NMN synthesis. These results indicate strong industrial potential for immobilized NMRK enzymes A-B in large-scale NMN production.

## Discussion conclusions and limitations

Chemical Synthesis of NMN employs various process routes. Using tetraacetylribose, benzoyl-β-D-ribose, and NAM as starting materials, ethyl nicotinate triacetyl nucleoside is synthesized. This is then subjected to ammonolysis, phosphorylation, glycosylation, and other chemical reactions to ultimately yield NMN. Although this synthesis process is relatively easy to control, the final product contains significant impurities. Separating these impurities and purifying the final product is difficult, and the overall conversion rate is low.Using triphenylmethyl-β-D-ribose as a starting material, bromination followed by substitution of bromine with nicotinamide, debenzoylation, and subsequent phosphorylation yields β-NMN with a total yield of approximately 31%.Using tetraacetylribose as a starting material gives a total yield of approximately 57%.This reaction process requires large amounts of organic solvents, causing a certain degree of environmental pollution. Furthermore, the condensation reaction of nicotinamide necessitates the use of the expensive promoter trimethylsilyl trifluoromethanesulfonate (TMSOTf), resulting in persistently high costs for the chemical synthesis of NMN [21]. Enzymatic Synthesis of NMN, on the other hand, utilizes the catalytic action of nicotinamide phosphoribosyltransferase (NAMPT). This pathway uses intracellular or extracellular sources of NAM or NR to generate NMN through the enzymatic activity of NAMPT or nicotinamide riboside kinase (NRK). This pathway is simple, involving fewer reaction steps, making it a highly suitable method for in vitro NMN synthesis. Studies have shown that using NAMPT derived from Vibriophage KVP40 and enhancing the NMN transport pathway in Escherichia coli achieved a record-breaking NMN yield of 16.2 g/L [22].

However, this method still faces the issue of requiring large quantities of multiple enzymes. Therefore, ensuring the high activity and high stability of the various biological enzymes used is key to achieving efficient NMN production. Enzyme immobilization is an important approach to improve enzyme reusability and enhance enzyme stability or resistance.

This text not only provides a method for enzyme immobilization but also describes a gene segment. Introducing this gene into host cells yields a genetically engineered strain that secretes higher amounts of the NRK enzyme protein. Using immobilized NRK enzyme to catalyze the synthesis of NMN from NR offers the following advantages:

Enhanced Stability and Reusability: The improved stability of the NMRK enzyme effectively prevents its activity from being inhibited by NR concentration. Simultaneously, the strengthened binding stability between the NMRK enzyme and the immobilization carrier prevents the enzyme from detaching during use. These combined effects enable the repeated, multiple uses of the NMRK enzyme (capable of over 10 consecutive cycles), increasing enzyme utilization efficiency and reducing NMN production costs. They also enhance batch-to-batch product consistency.

Simplified Purification & Higher Purity: The immobilized NMRK enzyme is easily separated from the reaction mixture, effectively preventing residual protein impurities in the product. Furthermore, the stable binding prevents the enzyme from detaching and introducing new impurities into the reaction system. This dual action simplifies the product purification process and significantly improves product purity.

Operational Flexibility for Continuous Processing: The immobilized NMRK enzyme possesses sufficient mechanical strength, allowing it to be used in stirred-tank reactors or packed into columns for substrate interaction. This facilitates the continuous and automated operation of the enzymatic reaction.

Compatible Reaction Conditions & Simplified Process: The immobilization procedure can be efficiently performed within a broad pH range of 5–9. Crucially, the synthesis of NMN from NR catalyzed by the immobilized NMRK enzyme operates optimally in the acidic environment (approximately pH 4–6) preferred by the substrate NR. Therefore, no significant pH adjustment is required during the NMN synthesis process, simplifying the catalytic reaction steps and facilitating large-scale industrial production of NMN.

This work establishes microcrystalline cellulose as an efficacious carrier for high-activity NMRK immobilization, advancing sustainable NMN biomanufacturing. To propel industrial translation, three research vectors emerge as critical: 1. Interfacial dynamics at carrier-enzyme junctions require atomic-resolution interrogation via synchrotron CD and cryo-EM, particularly to decode LX-1000NH/HFA inactivation pathways; 2. Operational robustness must be validated beyond 50 cycles under industrially relevant stressors—thermal gradients, substrate pulsing, and continuous-flow regimes; 3. Techno-economic modeling incorporating carrier recyclability (>95% retention target) and life-cycle energy metrics will delineate scalability thresholds. Such concerted efforts promise to transform immobilized enzyme design from empirical screening to mechanistic orchestration, ultimately enabling cell-free NAD precursor platforms.

## Supporting information

S1 File Data. (XLSX)
